# Research Advance on Qingfei Paidu Decoction in Prescription Principle, Mechanism Analysis and Clinical Application

**DOI:** 10.3389/fphar.2020.589714

**Published:** 2021-01-27

**Authors:** Wei Ren, Yue Ma, Raoqiong Wang, Pan Liang, Qin Sun, Qingrong Pu, Li Dong, Maryam Mazhar, Gang Luo, Sijin Yang

**Affiliations:** ^1^ National Traditional Chinese Medicine Clinical Research Base, Affiliated Traditional Chinese Medicine Hospital, Southwest Medical University, Luzhou, China; ^2^ Faculty of Integrative Medicine, Southwest Medical University, Luzhou, China

**Keywords:** qingfei paidu decoction, novel coronavirus pneumonia, prescription principle, mechanism analysis, clinical application

## Abstract

Since the sudden epidemic of coronavirus disease 2019 (COVID-19), the State Administration of Traditional Chinese Medicine immediately organized experts to formulate and screen the effective prescriptions of traditional Chinese medicine according to the characteristics of the novel coronavirus infection. Qingfei Paidu decoction (QFPDD) has been proven to be effective in multi-provincial clinical trials, and has been selected as a general prescription for the treatment of COVID-19 in different stages that was later promoted to be used nationwide. This review highlights the latest advances of QFPDD, focusing on the TCM theory, mechanism analysis, clinical application of QFPDD and its future perspectives. Moreover, an in-depth discussion of some valuable issues and possible development for future research on QFPDD is also discussed, aiming to provide a novel guide to combat the global epidemic COVID-19.

## 1 Introduction

As of November 1, 2020, novel coronavirus pneumonia (COVID-19), has spread over 211 countries around the world including all the continents, except Antarctica with around 46.43 million cumulative confirmed cases and 1.2 million deaths due to its strong infectiousness. The prevalence of COVID-19 has surpassed that of SARS in 2003, and is recognized as a severe health menace worldwide.

Since December 1, 2019, COVID-19 was emerged in Wuhan, Hubei province, China. Subsequently, the epidemic broke out throughout the country with the floating population during the Spring Festival. The mode of transmission for COVID-19 was soon recognized to be the inhalation of droplets from sneezing and coughing or the physical contact with the mucous secretions from infected individuals. People were generally susceptible and contracting the COVID-19 infection at exponentially high rate. Due to the sudden rise in the number of COVID-19 cases, China immediately launched the nationwide strict epidemic prevention and control guidelines. According to the Law of the People’s Republic of China on the Prevention and Treatment of Infectious Diseases, the COVID-19 epidemic is listed in class-B infectious disease while it is managed in accordance with Class A infectious diseases (Xue et al., 2020). Until now, the number of cases infected by COVID-19 continues to grow around the globe, and it is predicted to be continued for longer period of time (Guan et al., 2020). Still until now, proper and effective targeted therapy, drugs or vaccines, for COVID-19 epidemic control has not been identified. The accessibility of traditional drugs based on natural origin with effective therapeutic potential and the valuable historical treatment experience provide a more prominent therapeutic approach against COVID-19. Traditional Chinese medicine (TCM) has accumulated rich experience in the long-term practice of epidemic prevention and treatment, and it is characterized by broad-spectrum immunity, universal adaptability, foresight and so on. The unique advantages of TCM have attracted more and more attention to the epidemic prevention and treatment of COVID-19 (Yang et al., 2020a). Therefore, on January 27, 2019, the National Administration of Traditional Chinese Medicine launched the “Clinical screening for effective prescriptions of TCM for the prevention and treatment of pneumonia caused by novel coronavirus (2019-nCoV) infection” under the criteria “urgent, practical and effective”; nationwide. Qingfei Paidu Decoction (QFPDD), a multi-component herbal formula, was used clinically to treat 214 confirmed cases of COVID-19 with for three consecutive days as a course of treatment in four different pilot provinces in China from January 27, 2020 to February 5, 2020. The total effective rate was more than 90%, among them more than 60% of the cases showed significant improvement of symptoms and imaging manifestations and 30% of the patients showed stability of symptoms without aggravation or worsening (Yao et al., 2020). On February 18, 2020, the National Health Commission and National Administration of TCM jointly issued document No. 145 Diagnosis and Treatment Program of Novel Coronavirus Pneumonia (Trial Sixth edition). The document proposed to officially include TCM, QFPDD, in the clinical treatment of confirmed COVID-19 cases. QFPDD has been recommended as a general treatment prescription of TCM treatment for COVID-19 and has been promoted to the whole country for its remarkable clinical effect in the clinical prescription screening (Qin et al., 2020). Throughout the country, around 28 provinces, autonomous regions, and cities have been using this prescription, which is suitable for all periods and symptoms of COVID-19. Currently its use has been extended to treat suspected cases which has also been found effective and the feedback received is good.

QFPDD is formulated by the combination of syndrome differentiation and innovation based on the four classical prescriptions in *Treatise on Febrile Diseases* according to the pathogenic characteristics and development laws of COVID-19 (Jin, 2020). QFPDD has manifested its potential advantages and beneficial effects for the treatment of COVID-19. In this review, after summarizing the extant literature including CNKI, PubMed, Springer, Taylor & Francis, Google Scholar, and Baidu Scholar databases and other scientific resources e.g., Chinese Pharmacopoeia, 2020 edition, postgraduate research (PhD and MSc thesis, etc.), we have systematically summarized the TCM theory, modern mechanism analysis, clinical practice and application of QFPDD, hoping that it could offer some enlightenment for the further development and propel the research forward for efficiency, safety and controllable quality of QFPDD, so as to provide strong support for the global fight against the COVID-19 (Supplementary Figure).

### 1.1 TCM Theory and Prescription Principle of QFPDD

According to TCM theory, the experts have reached a consensus that COVID-19 belongs to a category of phytophthora blight (Li et al., 2020a), however, different experts have different understandings of COVID-19, including damp-toxin epidemic, cold-damp epidemic, and damp-heat epidemic. Wang and Miao et al. (2020) proposed COVID-19 as a damp-toxin epidemic caused by the damp toxin that belongs to yin, with the injury of *Yang* as the mainline (Miao et al., 2020; Wang et al., 2020c). Some believed that COVID-19 is a cold-damp epidemic caused by noxious dampness, and the basic pathogenesis is characterized by dampness, poison, blood stasis, and closure (Tong et al., 2020; Wang et al., 2020c; Xue et al., 2020). Luo and Zeng (2020) considered that COVID-19 is a damp-heat type caused by damp-heat epidemic toxin, and the main pathogenesis is the dampness, heat block of the Qi movement, endogenesis of phlegm and its transformation into fire and toxin, cremation of toxin and the combination of heat and blood stasis (Luo et al., 2020; Zeng and Sun, 2020).

Based on comprehensive analysis of COVID-19 clinical manifestations and syndrome types issued by the National Health Commission of PRC and various provinces in response to local conditions (Table 1), it is considered that COVID-19 is a damp-heat lung plague caused by damp-heat and epidemic toxin, and the pathogenesis and evolution process can be dry, and fire, and wind. At the very beginning, the pestilence attacks from the *Taiyang* meridian into the *Yangming* meridian quickly, or straight into the three *Yang* meridians, which is called *concurrent disease of three yang meridians*. But sometimes there is cold-dampness surrounding the exterior along with intense interior pathogenic fire. Or at the beginning, the exogenous pathogenic factors invade into three *Yin* meridians quickly, which may conduce to the syndrome of internal blockade and external collapse or syncope and collapse syndrome. The intermingled dampness and heat block the Qi movement, turbid phlegm hence appears inside and transforms into fire and toxin, intermingled heat and stasis is its main pathogenesis. Therefore, the treatment should be focused on dispelling dampness, heat and damp toxin, clearing dampness in triple warmer as well as strengthening vital Qi to eliminate pathogenic factors (Luo and Chen, 2020; Zhou, 2020). The most typical syndrome of COVID-19 is concurrent disease of three *Yang* meridians, which is often common in mild, moderate and part of severe cases. Hence, QFPDD is prescribed especially for this kind of syndrome.

**TABLE 1:** TCM Syndrome Types of COVID-19 in national and various provinces' prevention and control programs.

Countries and regions	Mild	Moderate/Ordinary	Severe	Critical	Convalescence
National health commission	Cold-dampness retention lung syndrome; damp-heat retention lung syndrome	Pathogenic dampness retention lung syndrome; cold-dampness stagnating the lung	Syndrome of epidemic toxin obstructing lung; syndrome of flaring heat in qifen and yingfen	Syndrome of internal blockade and external collapse	Lung and spleen qi deficiency, deficiency of both qi and yin
Hubei province	Syndrome of heat-toxin invading lung	Pathogenic dampness retention lung syndrome	Syndrome of accumulated dampness-toxicity	Syndrome of blazing heat-toxin	NA
Heilongjiang province	Damp warm retention lung syndrome	Phlegm-heat retention lung syndrome	Syndrome of pathogenic toxin obstructing lung	Syndrome of pathogenic toxin clouding orifices	Pathogenic factors residue, deficiency of both qi and yin
Beijing province	Syndrome of epidemic toxin invading lungs	NA	Epidemic toxin retention lung syndrome	Syndrome of epidemic toxin obstructing lung	Deficiency of both qi and yin
Shanghai province	Noxious dampness retention lung syndrome	NA	Syndrome of heat-toxin obstructing lung	Syndrome of internal blockade and external collapse	Lung and spleen qi deficiency, deficiency of both qi and yin
Guangdong province	Pathogenic dampness stagnating the lung, cardinal disadvantageous; syndrome of pathogenic heat congesting lung, impairment of the ascending and descending function of the lung	NA	Pathogenic heat obstructing lung syndrome, obstruction of fu-qi; warm-heat obstructing lung syndrome	Syndrome of internal blockade and external collapse	Pathogenic factors residue, deficiency of both qi and yin, deficiency of both lung and spleen
Jiangxi province	Noxious dampness retention lung syndrome, cardinal disadvantageous	Heat-toxin with dampness syndrome, impairment of the ascending and descending function of the lung	Syndrome of heat-toxin obstructing lung, obstruction of fu-qi	Syndrome of internal blockade and external collapse	NA
Shanxi province	Syndrome of exterior tightened by cold-dampness, impairment of fluid due to heat retention; syndrome of heat-toxin invading lung; external-cold and internal-heat	NA	Heat-toxin retention lung syndrome	Syndrome of internal blockade and external collapse	Syndrome of lingering heat, deficiency of both qi and yin
Tianjin province	Syndrome of heat-toxin invading lung	Pathogenic dampness retention lung syndrome	Syndrome of accumulated dampness-toxicity	Syndrome of blazing heat-toxin	NA
Yunnan province	Dampness-heat retention lung syndrome	Pathogenic heat retention lung syndrome	Syndrome of pathogenic toxin obstructing lung	Syndrome of internal blockade and external collapse	NA
Sichuan province	Wind heat with dampness syndrome; wind chill with dampness syndrome	Pathogenic dampness retention lung syndrome; dampness-heat retention lung syndrome	Pathogenic heat retention lung syndrome; epidemic toxin obstructing lung syndrome	Syndrome of internal blockade and external collapse	Pathogenic factors residue, deficiency of both qi and yin
Gansu province	Syndrome of warm pathogen attacking lung	Warm-heat retention lung syndrome	Syndrome of warm toxin obstructing lung	Syndrome of internal blockade and external collapse	NA

**FIGURE 1:**
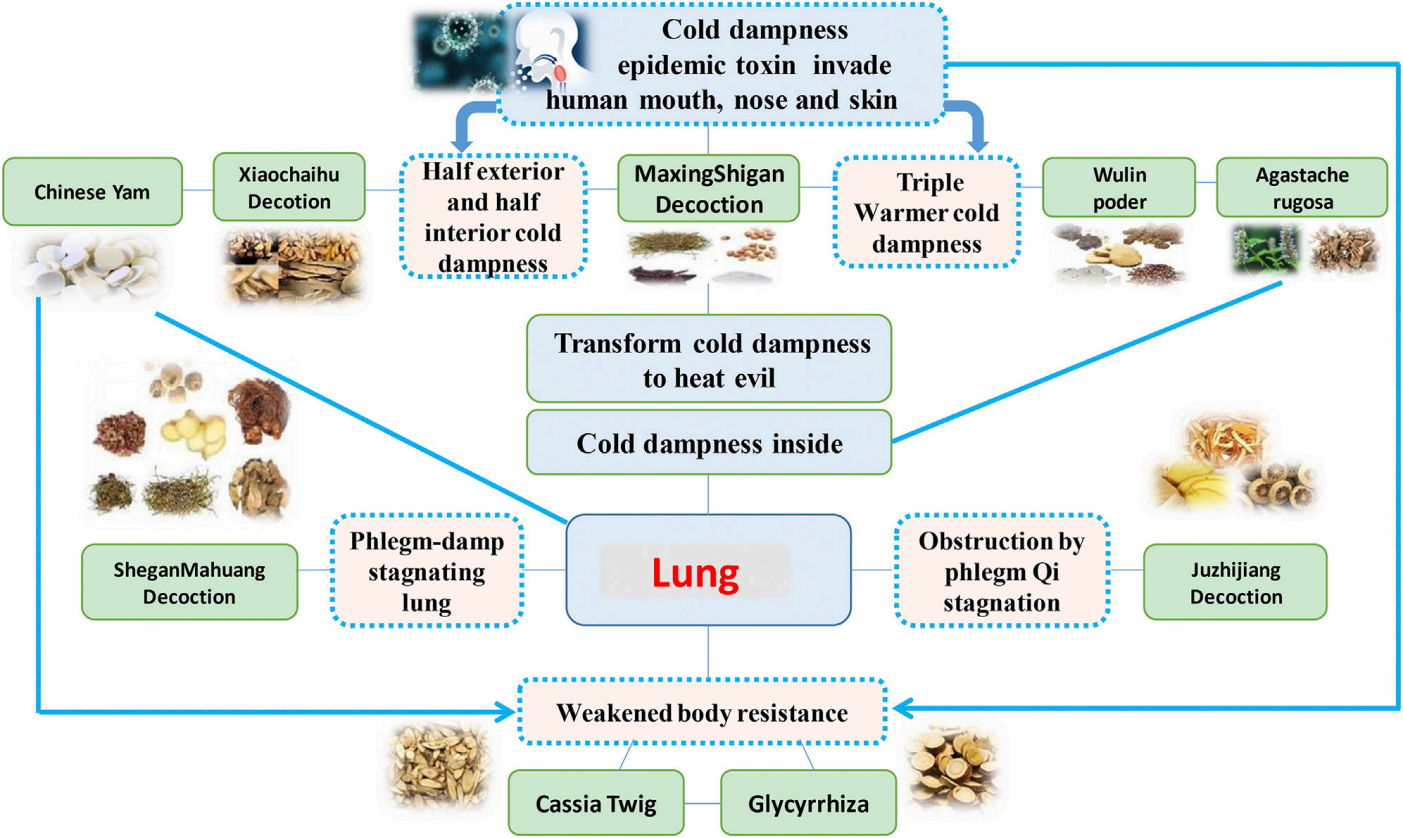
TCM theory and composition mechanism of durgs in QFPDD.

**FIGURE 2:**
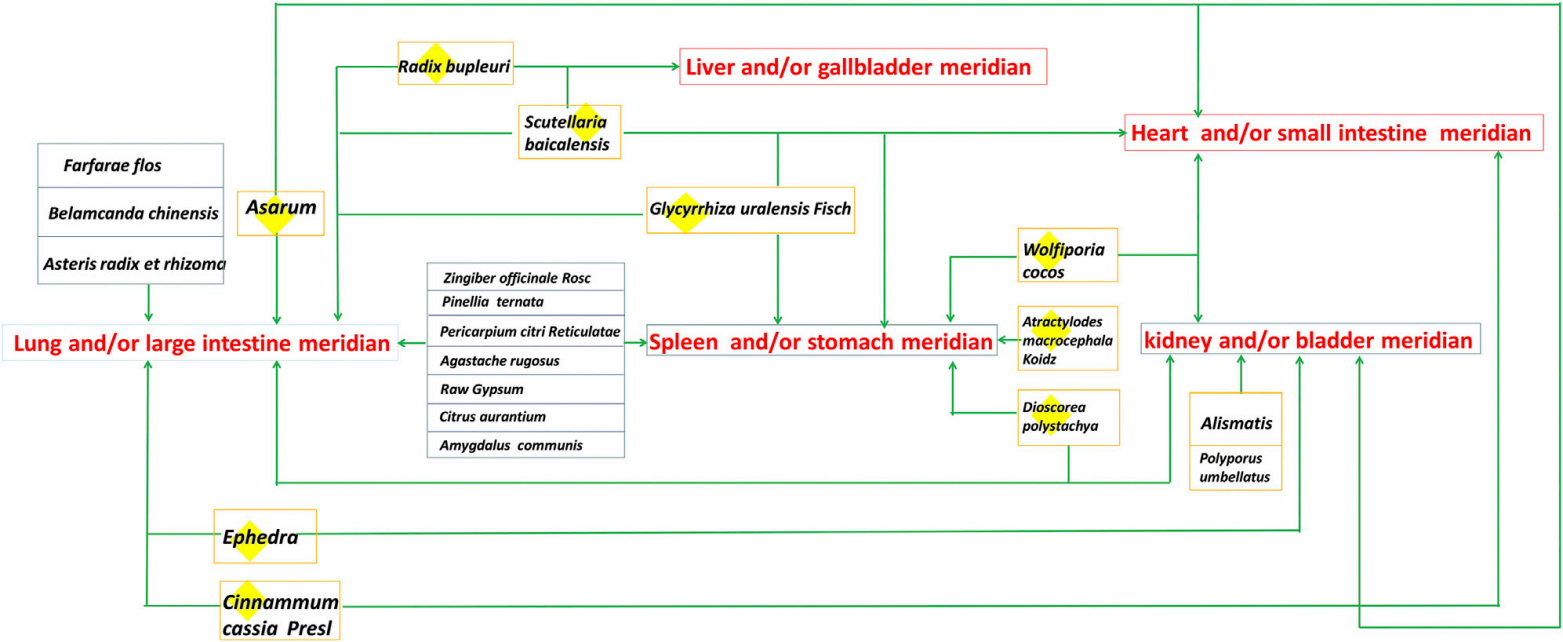
Drug‐meridian network of QFPDD.

**FIGURE 3:**
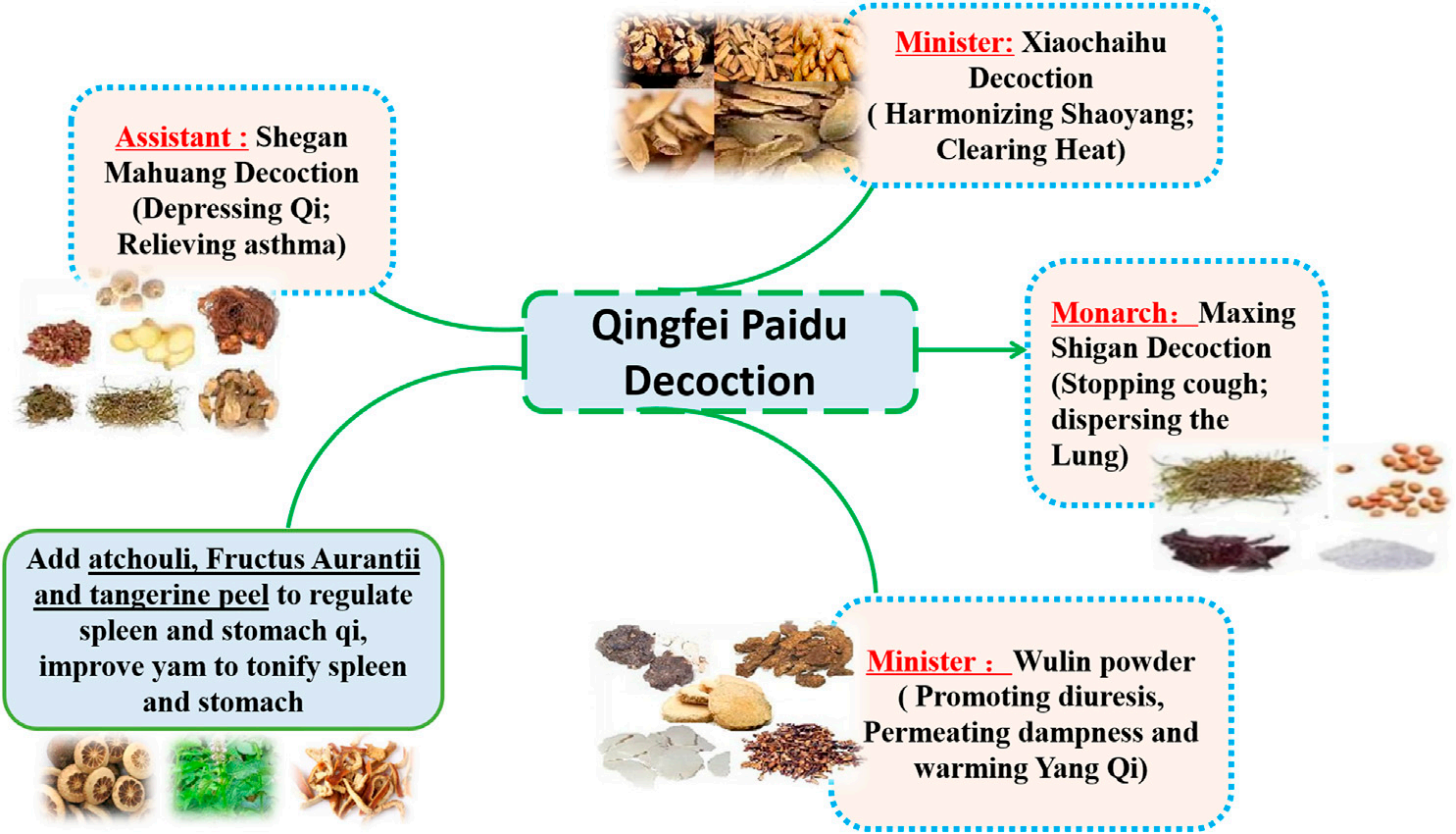
Compatibility of QFPDD in prescription.

QFPDD is composed of 21 TCMs, including *Herba Ephedrae* (*Ephedra sinica* Stapf*;* 9 g), *Radix Glycyrrhizae* (*Glycyrrhiza uralensis* Fisch.*;* 6 g*;* baked)*, Semen Armeniacae Amarum* (*Prunus armeniaca* L.*;* 9 g)*, Raw Gypsum* (15–30 g; first decocted)*, Ramulus Cinnamomi* (*Cinnamomum cassia* (L.) J.Presl*; *9 g)*, Rhizoma Alismatis* (*Alisma plantago-aquatica* Linn.*;* 9 g)*, Polyporus Umbellatus* (*Polyporus umbellaru* (Pers.) Fr.*;* 9 g)*, Rhizoma Atractylodis Macrocephalae* (*Atractylodes macrocephala* Koidz.*;* 9 g)*, Poria* (*Poria cocos* (Schw.) Wolf.*;* 15 g)*, Radix Bupleuri* (*Bupleurum chinensis* DC.*;* 16 g)*, Radix Scutellariae* (*Scutellaria baicalensis* Georgi*;* 6 g)*, Rhizome Pinelliae Preparata* (*Pinellia ternata* (Thunb.) Breit.*;* 9 g; processed with ginger)*, Rhizoma Zingiberis Recens* (*Zingiber officinale* Roscoe*;* 9 g)*, Radix Asteris* (*Aster tataricus* Linn. f.*;* 9 g)*, Flos Farfarae* (*Tussilago farfara* Linn.; 9 g)*, Rhizoma Belamcandae* (*Iris domestica* (L.) Goldblatt & Mabb.; 9 g)*, Herba Asari* (*Asarum sieboldii* Miq.; 6 g)*, Rhizoma Dioscoreae* (*Dioscorea oppositifolia* L.*;* 12 g)*, Fructus Aurantii Immaturus* (*Citrus sinensis* Osbeck; 6 g)*, Pericarpium Citri Reticulatae* (*Citrus aurantium* L.*;* 6 g)*,* and *Herba Pogostemonis* (*Pogostemon cablin (Blanco)* Benth.; 9 g). This prescription is mainly composed of Maxing Shigan decoction, Shegan Mahuang decoction, Xiaochaihu decoction and Wuling powder. In addition, it also incorporates Daqinglong decoction, Juzhijiang decoction, Fuling Xingren Gancao decoction, etc. QFPDD is a syncretic innovation of classical prescriptions from *Treatise on Febrile Diseases*, which act on different stages and viscera of water, dampness, phlegm, and fluid (Fan et al., 2020). This formula is suitable for the pathogenesis of COVID-19, affecting cold, dryness, damp toxin and dampness, and can effectively improve the symptoms. TCM theory and composition mechanism of QFPDD are summarized in Figure 1. The meridian tropisms of drugs in QFPDD are shown in Figure 2, where the top meridian tropism in QFPDD is lung meridian, indicating that drugs in QFPDD are mainly specific for lung diseases. The prescriptions of QFPDD are synergistic and complementary and the prescription principle of QFPDD is shown in Figure 3. Maxing Shigan decoction is to relieve exterior *Taiyang* syndrome, relieve superficies and ventilate lung Qi, clear heat and relieve panting; Shegan Mahuang decoction (*Fructus Jujubae* and *Fructus Schisandra chinensis* were taken out) is for lowering the adverse Qi and resolving fluid ventilate lung Qi, dispelling phlegm and relieving cough; Xiaochaihu decoction is for harmonizing half-superficies and half-interior *Shaoyang* syndrome, and large dose of raw gypsum is used to clear interior heat of the *Yangming* meridian, and Wuling powder is to warm the triple energizer and transform Qi and remove dampness by promoting diuresis; Juzhijiang decoction can activate Qi and dispel phlegm; *Herba Pogostemonis* can exorcise toxins and eliminate dampness; and *Rhizoma Dioscoreae* can strengthen the spleen and supplement the lung (Shen et al., 2020; Wang and Jin, 2020). The combination of *Ramulus Cinnamomi* and *Radix Glycyrrhizae* can nourish *Yang* and support healthy energy. QFPDD is not made up of drugs but multiple concordant prescriptions contributing to get twice the result with half the effort, so that the damp-heat and epidemic toxin can be quickly discharged (Wang et al., 2020a).

### 1.2 Mechanism Analysis of QFPDD

As described earlier, QFPDD contains a total of 21 TCMs, therefore, it is difficult to clearly explain the complex mechanisms of QFPDD in the treatment of COVID-19. Modern research on TCM holds that Chinese herbal compound formula plays an omnidirectional and overall regulatory role in the body due to the characteristics of multi-components, multi-targets and multi-path of the formula.

Recently, based on the reported components of QFPDD, several research groups have adopted the method of network pharmacology, molecular docking, and computer-aided drug design to provide data and clues for the multi-directional exploration of the material basis and pharmacodynamic mechanism of QFPDD in the treatment of COVID-19. Xu et al. (2020b) used the network pharmacology to screen significant effective compounds and key targets. Using TCMSP database, 148 related targets of 302 bioactive components in QFPDD were screened. Another database, GeneCards, using “COVID-19”, “2019-nCoV” and “Novel Coronavirus Pneumonia” as keywords, was used to screen 362 COVID-19 related targets where a total of 23 intersection targets were obtained by Venn analysis. By using the CentiScaPe plug-in of Cytoscape software, the network topology diagram of the 10 significant effective compounds, i.e., quercetin, luteolin, naringenin, kaempferol, beta-sitosterol, stigmasterol, baicalein, isorhamnetin, nobiletin, and wogonin (Table 2); and five pivotal targets, i.e., PTGS2, NOS2, PPARG, MAPK14, and PTGS1 were analyzed (Table 3). The results of molecular docking of the above most significant compound, quercetin, and target, PTGS2, with the highest degree value showed that the binding and interaction ability between these molecules was strong. The Gene Ontology (GO) and Kyoto Encyclopedia of Genes and Genomes (KEGG) pathway enrichment analysis of the key targets were done using the Cluster Profiler package of R software, which showed that significant compounds such as quercetin, luteolin, naringin, kaempferol, and baicalein have expectorant, antitussive, antiviral and anti-inflammatory effects in various degrees. The key targets were mainly concentrated in 144 related signaling pathways including IL-17, tuberculosis, human cytomegalovirus infection, TNF, MAPK, Hepatitis B, etc. (Table 4). It contained 28 biological effects including cytokine receptor binding, MAP kinase activity and phosphatase binding to regulate and control metabolism, immune regulation, lung function, inflammation, and other physiological processes (Xu et al., 2020b). Xu et al. (2020a) showed that 217 related targets of 186 active components and 200 COVID-19 related targets were screened, and 51 common drug-disease targets were obtained by Venn analysis. Then, five significantly effective compounds i.e., quercetin, luteolin, kaempferol, naringin, and isorhamnetin were obtained by using the CentiScaPe plug-in of Cytoscape software to further construct the network topology diagram. The GO and KEGG pathway enrichment analysis indicated that the key targets were mainly concentrated in 30 related signal pathways such as IL-17, NF-κB, TNF, MAPK, Th17, etc. It involved several biological functions such as inflammation, immune regulation, neuroprotection, reduction of lung injury, and other physiological processes (Xu et al., 2020a).

**TABLE 2:** The key active compounds of QFPDD.

Compounds	References	Compounds	References
Quercetin	Xu et al. (2020a), Zhou et al*.* (2020), Duan et al. (2020), Xu et al*.* (2020b), Wu et al., 2020b	Beta-sitosterol	Xu et al*.* (2020b), Wu et al., 2020b
Luteolin	Xu et al. (2020a), Zhou et al*.* (2020), Duan et al. (2020), Xu et al*.* (2020b), Wu et al., 2020b	Wogonin	Zhou et al*.* (2020), Duan et al*.* (2020), Xu et al*.* (2020b)
Kaempferol	Xu et al. (2020a), Zhou et al*.* (2020), Duan et al. (2020), Wu et al., 2020b, Xu et al*.* (2020b)	Baicalein	Xu et al*.* (2020b)
Naringenin	Xu et al. (2020a), Zhou et al*.* (2020), Duan et al. (2020), Xu et al. (2020a), Xu et al*.* (2020b)	Nobiletin	Xu et al*.* (2020b)
Isorhamnetin	Xu et al. (2020a), Xu et al*.* (2020b)	Stigmasterol	Xu et al*.* (2020b)

**TABLE 3:** The main key targets of QFPDD in the treatment of COVID-19.

Key targets	References	Key targets	References
Cell tumor antigen p53 (TP53)	Peng et al. (2020), Duan et al. (2020), Zhou et al. (2020)	Caspase 3 (CASP3)	Peng et al. (2020), Xu et al. (2020a), Yan et al. (2020), Duan et al. (2020), Zhou et al. (2020)
Protein kinase B1(Akt1)	Peng et al. (2020), Wu et al., 2020b	Janus kinase 2 (JAK2)	Peng et al. (2020)
Nuclear factor nuclear transcription factor-κB p105 subunit (NFKB1)	Peng et al. (2020)	Nuclear factor transcription factor-κB p100 subunit (NFKB2)	Peng et al. (2020)
Nuclear factor p65 subunit (RELA)	Peng et al. (2020)	Calmodulin 1 (CALM1)	Peng et al. (2020), Xu et al. (2020a)
Adenylate cyclase type 1 (ADCY1)	Peng et al. (2020)	Eukaryotic translation initiation factor 2, subunit 3 (EIF2S3)	Peng et al. (2020)
Adenylate cyclase type 2 (ADCY2)	Peng et al. (2020)	B-cell CLL/lymphoma 2 (BCL2)	Peng et al. (2020), Xu et al. (2020a), Zhou et al. (2020)
Heat shock protein α A1 (HSP90AA1)	Peng et al. (2020)	Protein kinase C-delta (PRKCD)	Peng et al. (2020)
Adenylate cyclase type 5 (ADCY5)	Peng et al. (2020)	Jun proto-oncogene (JUN)	Peng et al. (2020), Wu et al., 2020b
Recombinant human glucocorticoid receptor (NR3C1)	Peng et al. (2020)	Prostaglandin-endoperoxide synthase 2 (PTGS2)	Xu et al. (2020a), Xu et al*.* (2020b), Duan et al. (2020), Zhou et al. (2020)
Mitogen-activated protein kinase 8 (MAPK8)	Xu et al. (2020a), Duan et al. (2020), Zhou et al. (2020), Wu et al., 2020b	Prostaglandin-endoperoxide synthase 1 (PTGS1)	Xu et al. (2020a), Xu et al*.* (2020b), Zhou et al. (2020)
Mitogen-activated protein kinase 3 (MAPK3)	Peng et al. (2020), Xu et al. (2020a), Zhou et al. (2020)	Dipeptidyl peptidase-4 (DPP4)	Xu et al. (2020a), Yan et al. (2020)
Human NK-κB inhibited protein α (NFKBIA)	Peng et al. (2020)	V-rel reticuloendotheliosis viral oncogene homolog A (RELA)	Xu et al. (2020a), Zhou et al. (2020)
Bcl2-associated X protein (BAX)	Xu et al. (2020a), Zhou et al. (2020)	V-fos FBJ murine osteosarcoma viral oncogene homolog (FOS)	Xu et al. (2020a), Zhou et al. (2020)
Apolipoprotein D (APOD)	Xu et al. (2020a)	Lymphocyte specific tyrosine kinase (LCK)	Peng et al. (2020)
Peroxisome proliferative activated receptor, gamma (PPARG)	Xu et al. (2020a), Xu et al*.* (2020b), Zhou et al. (2020)	Signal transducerand activator of transcription 1(STAT1)	Xu et al. (2020a), Zhou et al. (2020)
Nitric oxide synthase (NOS2)	Xu et al. (2020a), Xu et al*.* (2020b), Zhou et al. (2020)	Retinoblastoma 1 (RB1)	Xu et al. (2020a), Duan et al. (2020), Zhou et al. (2020)
Mitogen-activated protein kinase 14 (MAPK14)	Xu et al. (2020a), Xu et al*.* (2020b), Duan et al. (2020), Zhou et al. (2020)	Interleukin-6 (IL-6)	Xu et al. (2020a), Zhou et al. (2020), Wu et al., 2020b
Heme oxygenase (decycling) 1 (HMOX1))	Xu et al. (2020a), Duan et al. (2020), Zhou et al. (2020)	Apoptosis-related cysteine peptidase (CASP8)	Xu et al. (2020a), Zhou et al. (2020)
Intercellular adhesion molecule 1 (ICAM1)	Xu et al. (2020a), Duan et al. (2020), Zhou et al. (2020)Z	Superoxide dismutase 1 (SOD1)	Xu et al. (2020a), Zhou et al. (2020)
Epidermal growth factor receptor (EGFR)	Xu et al. (2020a), Duan et al. (2020), Zhou et al. (2020)	Protein kinase C alpha type (PRKCA)	Xu et al. (2020a), Zhou et al. (2020)
Bcl-2-like protein 1 (BCL2L1)	Xu et al. (2020a), Zhou et al. (2020)	Heat shock 70 kDa protein 5 (HSPA5)	Xu et al. (2020a), Zhou et al. (2020)
Mitogen-activated protein kinase 1 (MAPK1)	Xu et al. (2020a), Duan et al. (2020), Wu et al., 2020b	Interleukin-1β (IL-1β)	Xu et al. (2020a), Duan et al. (2020)
Chemokine (C-C motif) ligand 2 (CCL2)	Xu et al. (2020a), Duan et al. (2020), Zhou et al. (2020)	Protein kinase C beta type (PRKCB)	Xu et al. (2020a), Zhou et al. (2020)
Serine protease inhibitor protein E1 (SERPINE1)	Xu et al. (2020a), Zhou et al. (2020)	Nitric oxide synthase 3 (NOS3)	Xu et al. (2020a), Zhou et al. (2020)
Interleukin-2 (IL-2)	Xu et al. (2020a), Zhou et al. (2020)	Heat shock 27 kDa protein 1 (HSPB1)	Xu et al. (2020a), Zhou et al. (2020)
Interleukin-1α (IL-lα)	Xu et al. (2020a), Zhou et al. (2020)	Poly ADP-ribose polymerase 1 (PARP1)	Xu et al. (2020a), Zhou et al. (2020)
Chemokine CXC motif ligand 2 (CXCL2)	Xu et al. (2020a), Zhou et al. (2020)	Chemokine CXC motif ligand 11 (CXCL11)	Xu et al. (2020a), Zhou et al. (2020)
C-reactive protein (CRP)	Xu et al. (2020a), Zhou et al. (2020)	Chemokine CXC motif ligand 10 (CXCL10)	Xu et al. (2020a), Zhou et al. (2020)
CD40 ligand (CD40LG)	Xu et al. (2020a), Zhou et al. (2020)	BCL2-antagonist of cell death (BAD)	Xu et al. (2020a), Zhou et al. (2020)
Interferon regulatory factor 1 (IRF1)	Xu et al. (2020a), Zhou et al. (2020)	Catalase (CAT)	Xu et al. (2020a), Duan et al. (2020), Zhou et al. (2020)
Phospholipase A2 (PLA2G4A)	Xu et al. (2020a), Zhou et al. (2020)	cAMP responsive element binding protein 1 (CREB1)	Xu et al. (2020a), Zhou et al. (2020)
Cyclin D3 (CCND3)	Xu et al*.* (2020a)	Myeloid cell leukemia sequence 1 (MCLl)	Xu et al. (2020a)
Interleukin-4 (IL-4)	Xu et al. (2020a), Duan et al. (2020), Zhou et al. (2020)	Cyclin-dependent kinase 4 (CDK4)	Xu et al. (2020a), Zhou et al. (2020)
Angiotensin I-converting enzyme (ACE)	Yan et al. (2020)	Glucose-6-phosphate dehydrogenase (G6PD)	Xu et al. (2020a), Zhou et al. (2020)
Angiotensin I-converting enzyme 2 (ACE2)	Yan et al. (2020)	Furin (FURIN)	Yan et al. (2020)
Angiotensin II type 1 receptor (AT1R/AGTR1)	Yan et al. (2020)	Caspase 6 (CASP6)	Yan et al. (2020)
Myeloid cell Leukemia sequence 1 (MCL1)	Yan et al. (2020), Zhou et al. (2020)	Polymerase (DNA directed), delta 1, catalytic subunit 125 kDa (POLD1)	Yan et al. (2020)
Tumor necrosis factor (TNF)	Yan et al. (2020), Zhou et al. (2020)	Interleukin- 10 (IL-10)	Duan et al. (2020), Zhou et al. (2020)
Interferon, gamma (IFNG)	Duan et al. (2020), Zhou et al. (2020)	Interleukin-8 (IL-8)	Duan et al. (2020), Zhou et al. (2020)
Transforming growth factor, beta 1 (TGFB1)	Zhou et al. (2020)		

**TABLE 4:** Main enriched signaling pathways of QFPDD in the treatment of COVID-19.

Pathway name	References	Pathway name	References
Adherens junction	Zhao et al. (2020)	AGE-RAGE signaling pathway in diabetic complications	Xu et al. (2020b), Xu et al*.* (2020a)
Focal adhesion	Zhao et al. (2020)	C-type lectin receptor signaling pathway	Xu et al. (2020b), Xu et al*.* (2020a)
Osteoclast differentiation	Zhao et al. (2020), Xu et al. (2020b), Wu et al., 2020b	HIF-1 signaling pathway	Xu et al. (2020b), Xu et al*.* (2020a), Wu et al., 2020b
Estrogen signaling pathway	Zhao et al*.* (2020)	Toxoplasmosis	Xu et al. (2020b), Wu et al., 2020b, Xu et al*.* (2020a), Duan et al*.* (2020)
Thyroid hormone signaling pathway	Zhao et al*.* (2020), Wu et al., 2020b	*Yersinia* infection	Xu et al. (2020b)
Relaxin signaling pathway	Zhao et al*.* (2020)	Hepatitis B	Xu et al. (2020b), Xu et al*.* (2020a), Wu et al., 2020b
Prolactin signaling pathway	Zhao et al*.* (2020), Wu et al., 2020b	NOD-like receptor signaling pathway	Xu et al. (2020b), Wu et al., 2020b, Duan et al*.* (2020), Zhou et al*.* (2020)
Oxytocin signaling pathway	Zhao et al*.* (2020)	Kaposi sarcoma-associated herpesvirus infection	Xu et al. (2020b), Xu et al*.* (2020a)
Glucagon signaling pathway	Zhao et al*.* (2020)	Pertussis	Xu et al. (2020b), Wu et al., 2020b, Xu et al*.* (2020a), Duan et al*.* (2020)
Th17 cell differentiation	Zhao et al*.* (2020), Xu et al. (2020b)	Leishmaniasis	Xu et al. (2020b), Wu et al., 2020b, Xu et al*.* (2020a), Duan et al*.* (2020)
B cell receptor signaling pathway	Zhao et al*.* (2020), Wu et al., 2020b	Endocrine resistance	Xu et al. (2020b)
T cell receptor signaling pathway	Zhao et al*.* (2020), Wu et al., 2020b	FoxO signaling pathway	Xu et al. (2020b), Wu et al., 2020b, Duan et al*.* (2020)
Neurotrophin signaling pathway	Zhao et al*.* (2020)	Prion diseases	Xu et al. (2020b)
Dopaminergic synapse	Zhao et al*.* (2020)	Pancreatic cancer	Wu et al., 2020b, Xu et al. (2020b)
ErbB signaling pathway	Zhao et al*.* (2020), Wu et al., 2020b	Hepatitis C	Wu et al., 2020b, Duan et al*.* (2020)
MAPK signaling pathway	Zhao et al*.* (2020), Duan et al*.* (2020), Zhou et al*.* (2020)	Ras signaling pathway	Wu et al., 2020b
PI3K-Akt signaling pathway	Zhao et al*.* (2020), Xu et al. (2020b), Wu et al., 2020b	Bladder cancer	Wu et al., 2020b
TNF signaling pathway	Zhao et al*.* (2020), Wu et al., 2020b, Xu et al. (2020b), Duan et al*.* (2020), Zhou et al*.* (2020)	Prostate cancer	Wu et al., 2020b
Wnt signaling pathway	Zhao et al*.* (2020)	Melanama	Wu et al., 2020b
VEGF signaling pathway	Zhao et al*.* (2020), Xu et al. (2020b), Wu et al., 2020b	Thyroid hormone signaling pathway	Wu et al., 2020b
Ribosome	Zhao et al*.* (2020)	Chronic myeloid leukemia	Wu et al., 2020b
IL-17 signaling pathway	Xu et al. (2020b), Xu et al*.* (2020a)	Glioma	Wu et al., 2020b
Chagas disease (American trypanosomiasis)	Xu et al. (2020b), Wu et al., 2020b, Xu et al*.* (2020a), Duan et al*.* (2020)	Endometrial cancer	Wu et al., 2020b
Tuberculosis	Xu et al. (2020b), Wu et al., 2020b, Xu et al*.* (2020a), Duan et al*.* (2020)	Influenza A	Wu et al., 2020b, Xu et al. (2020b), Duan et al*.* (2020)
Human cytomegalovius infection	Xu et al. (2020b), Xu et al*.* (2020a)	Toll-like receptor signaling pathway	Wu et al., 2020b, Xu et al*.* (2020a), Duan et al*.* (2020), Zhou et al*.* (2020), Yang et al. (2020a)
Epithelial cell signaling in helicobacter pylori infection	Wu et al., 2020b	*Salmonella* infection	Wu et al., 2020b, Duan et al*.* (2020)
Melanoma	Wu et al., 2020b	Colorectal cancer	Wu et al., 2020b
RIG-I-like receptor signaling pathway	Wu et al., 2020b	Small cell lung cancer	Wu et al., 2020b
Herpes simplex infection	Wu et al., 2020b, Duan et al*.* (2020)	Non-alcoholic fatty liver disease	Wu et al., 2020b
Shigellosis	Wu et al., 2020b	HTLV-I infection	Wu et al., 2020b
Cytosolic DNA-sensing pathway	Wu et al., 2020b	Apoptosis	Xu et al*.* (2020a)
Acute myeloid leukemia	Wu et al., 2020b	Human immunodeficiency virus 1 infection	Xu et al*.* (2020a)
Measlea	Xu et al*.* (2020a)	Proteoglycans in cancer	Wu et al., 2020b
Non-small cell lung cancer	Wu et al., 2020b	Glutamatergic synapse	Jin et al*.* (2020)
Amphetamine addiction	Jin et al*.* (2020)	Long-term potentiation	Jin et al*.* (2020)
Long-term depressio	Jin et al*.* (2020)	Retrograde endocannabinoid signaling	Jin et al*.* (2020)
Cocaine addiction	Jin et al*.* (2020)	Nitrogen metabolism	Jin et al*.* (2020)
Nicotine addiction	Jin et al*.* (2020)	Neuroactive ligand-receptor interaction	Jin et al*.* (2020), Chen et al. (2020a)
Interleukin-4 and interleukin-13 signaling	Peng et al*.* (2020)	Interleukin-1 processing	Peng et al*.* (2020)
Adrenoceptors	Peng et al*.* (2020)	IκBα variant leads to EDA-ID	Peng et al*.* (2020)
CLEC7A/inflammasome pathway	Peng et al*.* (2020)	DEx/H-box helicases activate type I IFN and inflammatory cytokines production	Peng et al*.* (2020)
G alpha (s) signaling events	Peng et al*.* (2020)	G alpha(z) signaling events	Peng et al*.* (2020)
Tp53 regulates transcription of DNA repair	Peng et al*.* (2020)	RIP-mediated NF-κB activation via ZBP1	Peng et al*.* (2020)
Interleukin-21 signaling	Peng et al*.* (2020)	PI5P, PP2A and IER3 regulate PI3K/Akt signaling	Peng et al*.* (2020)
Interleukin-2 signaling	Peng et al*.* (2020)	Signaling by SCF-KIT	Peng et al*.* (2020)
Erythropoietin activatesPphosphoinositide-3-kinase (PI3K)	Peng et al*.* (2020)	Activation of the AP-1 family of transcription factors	Peng et al*.* (2020)
Interleukin-10 signaling	Peng et al*.* (2020)	Interleukin receptor SHC signaling	Peng et al*.* (2020)
Adenylate cyclase inhibitory pathway	Peng et al*.* (2020)	Calmodulin induced events	Peng et al*.* (2020)
Inflammatory bowel disease (IBD)	Duan et al*.* (2020)	Cytokine-cytokine receptor interaction	Duan et al*.* (2020), Zhou et al*.* (2020)
Rheumatoid arthritis	Duan et al*.* (2020)	Amebiasis	Duan et al*.* (2020)
African trypanosomiasis	Duan et al*.* (2020)	Malaria	Duan et al*.* (2020)
Dteroid biosynthesis	Chen et al. (2020a)	PPAR signaling pathway	Chen et al. (2020a)
Adipocytokine signaling pathway	Chen et al. (2020a)	Steroid hormone biosynthesis	Chen et al. (2020a)

Zhao et al*.* revealed that 464 compounds of QFPDD corresponded to 790 different putative targets, of which 232 targets were co-expressed with angiotensin-converting enzyme 2 (ACE2), the receptor of 2019-nCoV. Main signaling pathways regulated by key targets of QFPDD are shown in Table 3, where the main targets are concentrated on two types of disease pathways i.e., virus infection and lung injury. In addition, 48 important targets interacted densely with six proteins of HIV, indicating its potential antiviral effect. Key targets regulated a series of signaling pathways in biological processes such as endocrine system, immune system, translation, nervous system, and signal transduction (Zhao et al., 2020).

Wu et al. (2020b) showed that the QFPDD compound-pneumonia target network contained 292 compounds and 214 corresponding potential targets and the top five pivotal targets were AKT serine/threonine kinase 1 (AKT1), interleukin-6 (IL-6), mitogen-activated protein kinase 8 (MAPK8), mitogen-activated protein kinase 1 (MAPK1), and jun proto-oncogene (JUN). The GO and KEGG enrichment analysis and screening yielded 122 related signaling pathways, including non-small cell lung cancer, small cell lung cancer, hypoxia inducible factor-1, toll-like receptor signaling pathway, T cell receptor signaling pathway and other pathways related to pneumonia. Moreover, the same enrichment analysis also included TNF signaling pathway, P13k-Akt signaling pathway, MAPK signaling pathway, B cell receptor signaling pathway, apoptosis, and other pathways related to the reduction of lung injury (Table 4). The molecular docking results indicated that some core compounds such as ergosterol, shionone, tussilagone, etc. of the TCMs present in QFPDD had a certain degree of binding activity for 2019-nCoV 3C-like protease (3CLpro) and ACE2. It was worthwhile pointing out that ergosterol is the only one that can form a hydrogen bond with 3CLpro of 2019-nCoV (Wu et al., 2020b). In another study by Yan et al. (2020) QFPDD compound-2019-nCoV and COVID-19 target-biological function network was screened, it contained 163 active ingredients, 10 protein targets, and 42 biological functions such as renin-angiotensin regulation of blood volume and systemic arterial blood pressure to treat COVID-19. The results of preliminary molecular docking showed that the core ingredients had a good affinity with SARS-CoV-2 3CL hydrolase to form complexes with stable conformations and high binding energy, indicating that QFPDD might treat COVID-19 through RAS signaling pathway (Yan et al., 2020). Cytokine storm is considered one of the central causes of clinical sudden deterioration of COVID-19. It has been reported that QFPDD had an inhibitory effect on cytokine storm in the treatment of COVID-19 by acting on multiple targets and pathways with multiple components (Zhou et al., 2020). Duan et al. (2020) revealed that QFPDD had a potential common action mechanism in the treatment of SARS, MERS, and COVID-19. 337 corresponding targets of 246 components in QFPDD and 148 common disease-related targets for SARS, MERS, and COVID-19 were screened, and 44 common drug-disease targets were obtained by Venn analysis. The GO and KEGG pathway enrichment analysis of the key targets indicated that the key targets were mainly concentrated in 77 related signal pathways such as pertussis, tuberculosis, MAPK, FoxO, TNF, NOD-like receptor signaling pathways, and other pathways related to viral pneumonia. biological angiogenesis, immune response, nitric oxide synthesis and cell apoptosis might be the potential common mechanisms of QFPDD in the treatment of SARS, MERS, and COVID-19 (Duan et al., 2020).

In addition, Peng et al. (2020) constructed the interaction network of *Formula-Herb-Disease-Targets-Pathways* based on the three main clinical symptoms of COVID-19: pneumonia, fever, and cough. The research results indicated that key-targets such as cell tumor antigen p53 (tp53), protein kinase B1 (Akt1), nuclear factor nuclear transcription factor-κB (NK-κB) p105 subunit (NFKB1), nuclear factor p65 subunit (RELA), human NK-κB inhibited protein α (NFKBIA), etc. were mainly related to the regulation of apoptosis and immune response, inflammatory response, improving lung function, etc. The GO and KEGG pathway enrichment analysis indicated that the 103 key targets were mainly concentrated in the signal pathways such as interleukin signaling, adrenoceptors, seven members of the family of c-type lectin domains A (CLEC7A)/inflammasome pathway, phosphoinositide-3-kinase (PI3K)/protein kinase B (Akt) inflammatory signaling pathway, tp53 regulates transcription of DNA repair, etc. which might be the main pathways related to QFPDD’s effect on the treatment of COVID-19 accompany with lung injury, fever, cough, and other symptoms (Peng et al., 2020).

Based on computer-aided drug design, Jin et al. (2020) systematically explored and analyzed the material basis and molecular mechanism of QFPDD in the three aspects of detoxification, anti-inflammatory storm, and diuresis-removing dampness. Molecular docking virtual screening was performed based on the 2,740 compounds in QFPDD and the targets including ACE2, interleukin-6 receptor (IL-6R), and aquaporins (APQ). The mechanism of action was predicted by reverse target prediction, GO and KEGG pathway enrichment analysis for *Atractylodes macrocephala, Polyporus umbellatus, Poria cocos, and Alisma plantago-aquatica*. Research showed that Xiaochaihu decoction ranked the first in the number of potentially active compounds to block the virus and suppress inflammatory storm among the five classic prescriptions of QFPDD. The top three most prominent drugs to block the key binding sites of the virus were *Radix Glycyrrhizae, Herba Ephedrae*, and *Citrus aurantium*, while the top three to suppress inflammatory storm were *Radix Glycyrrhizae, Radix Asteris, and Radix Bupleuri*. Quercetin and its derivatives, the potential dual-target active compounds, had a high binding ability to ACE2 and IL-6R targets. *Atractylodes macrocephala, Cinnamomum cassia, Poria cocos, Polyporus umbellatus, and Alisma plantago-aquatica* lacked compounds that blocked viruses and suppressed inflammatory storms, but dehydroeburicoic acid, scopoletin, alismoxide and alpha-D-galactose contained in the above drugs had the potential binding ability with AQP4. Each component of the sub-medical prescription is reasonably compatible and plays a role in prevention and treatment through multi-point cooperation and complementary advantages. The interaction between these targets can form a molecular network, and it is found that many active components of QFPDD play a role in virus invasion, virus replication, and multiple organ damage (Jin et al., 2020). Furthermore, Chen et al. (2020a) divided QFPDD into five functional units (four sub-medical prescriptions and the rest) in the light of the compatibility theory of TCM. Results showed that all the five functional units had a positive effect on COVID-19 independently, and it involved physiological processes such as inflammation, bacterial and viral responses, immune system, signaling transduction, etc (Chen et al., 2020a). Yang et al. (2020c) also reported the chemical composition and pharmacological mechanism of QFPDD which indicated the thrombin and Toll-like receptor (TLR) signaling pathway were suggested to be main pathways for Maxing Shigan decoction mediated anti-inflammatory effects (Yang et al., 2020c).

### 1.3 *In Vivo* Distribution and Metabolomics of QFPDD

Liu et al. (2020b) investigated the main chemical constituents in QFPDD and the tissues distribution of the main absorbed constituents in mice following oral administration of QFPDD. As shown in Table 5, a total of 39 compounds were identified from QFPDD using UHPLC-Q-Orbitrap HRMS. After administered QFPDD in mice (2.6 g/100 g, ig), 12, 9, 10, 8, 9, and 10 constituents were identified in serum, heart, lung, spleen, liver, and kidney, respectively. The results showed that these nine constituents (ephedrine, pseudoephedrine, amygdalin, prunasin, liquiritin, hyperoside, hesperidin, baicalin, and risflorentin) could be quickly absorbed into the circulation system and then widely distributed in various tissues. At 0.5 h, except baicalin, the exposure of the other eight target components reached a peak in serum and tissues. The exposure of baicalin was peaked at 2 or 4 h. At 0.5 h, the exposure of target components to lung tissue was ranked as follows: ephedrine (2,759.11 ± 784.39 ng/g), prunasin (1819.7 ± 427.28 ng/g), pseudoephedrine (880.6 ± 287.97 ng/g), amygdalin (304.43 ± 234.7 ng/g), hesperidin (78.33 ± 38.38 ng/g), risflorentin (8.62 ± 4.66 ng/g), baicalin (8.53 ± 1.91 ng/g), hyperoside (7.72 ± 1.63 ng/g), liquiritin (7.68 ± 5.19 ng/g). At 2 h, ephedrine (776.61 ± 148.4 ng/g), prunasin (173.77 ± 58.21 ng/g), pseudoephedrine (84.68 ± 59.04 ng/g), baicalin (49.33 ± 17.06 ng/g), amygdalin (1.26 ± 0.26 ng/g) (Liu et al., 2020b). Furthermore, Wu et al. (2020a) indicated that treatment with QFPDD (1.5, 6 g/kg/day, p.o.) for continued 5 days, could significantly regulate the host metabolism and gut microbiota composition in rats such as enriched romboutsia, turicibacter, and *clostridium*_sensu_stricto_1, and decreased norank_f_Lachnospiraceae. The results from GC-MS and LC-MS/MS identified a total of 23 and 43 differential metabolites respectively that were altered by QFPDD. The metabolic pathways of these differential metabolites included glycerophospholipid metabolism, linoleic acid metabolism, TCA cycle, and pyruvate metabolism (Wu et al., 2020a).

**TABLE 5:** Components of QFPDD distribution in the organs.

Name	CAS No	Distribution					
		Serum	Liver	Heart	Spleen	Lung	Kidney
Synephrine	94-07-5	－	－	－	－	－	－
Dihydroxyacetone	96-26-4	－	－	－	－	－	－
Gallic acid monohydrate	5,995-86-8	－	－	－	－	－	－
Neochlorogenic acid	906-33-2	－	－	－	－	－	－
(1R,2S)-2-(Methylamino)-1-phenylpropan-1-ol	299-42-3	＋	＋	＋	＋	＋	＋
Pseudoephedrine	90-82-4	＋	＋	＋	＋	＋	＋
Caffeic acid	331-39-5	－	－	－	－	－	－
Chlorogenic acid	327-97-9	－	－	－	－	－	－
Cryptochlorogenic acid	905-99-7	－	－	－	－	－	－
(R)-amygdalin	29,883-15-6	＋	＋	＋	＋	＋	＋
Benzeneacetonitrile, a-(b-D-glucopyranosyloxy)-, (aR)-	99-18-3	＋	＋	＋	＋	＋	＋
(-)-3,5-Dicaffeoyl quinic acid	89,919-62-0	－	－	－	－	－	－
Ferulic acid	1,135-24-6	－	－	－	－	－	－
Liquiritin	551-15-5	＋	＋	＋	＋	＋	＋
Isochlorogenic acid B	14,534-61-3	－	－	－	－	－	－
3,5-Dicaffeoylquinic acid	2,450-53-5	＋	－	－	－	＋	＋
Hyperoside	482-36-0	＋	＋	＋	－	＋	＋
Rutin	153-18-4	－	－	－	－	－	－
Resveratrol	501-36-0	－	－	－	－	－	－
Naringen	4,493-40-7	－	－	－	－	－	－
Hesperiden	520-26-3	＋	＋	＋	＋	＋	＋
Isochlorogenic acid C	57,378-72-0	－	－	－	－	－	－
Cinnamaldehyde	14,371-10-9	－				－	－
Baicalin	21,967-41-9	＋	＋	＋	＋	＋	＋
Quercetin	117-39-5	－	－	－	－	－	－
Luteolin	491-70-3	－	－	－	－	－	－
Kaempferol	520-18-3	－	－	－	－	－	－
Irisflorentin	41,743-73-1	＋	＋	＋	＋	＋	＋
Gingerol	23,513-14-6	－	－	－	－	－	－
2,5,7-trimethoxyphenanthren-3-ol	51,415-00-0	－	－	－	－	－	－
Asarinin	133-04-0	－	－	－	－	－	－
Glycyrrhizic acid	1,405-86-3	＋	－	－	－	－	－
(8)-Gingerol	23,513-08-8	－	－	－	－	－	－
Atractylenolide I	73,069-13-3	－	－	－	－	－	－
Saikosaponin A	20,736-09-8	－	－	－	－	－	－
Tussilagone	104012-37-5	－	－	－	－	－	－
(10)-Gingerol	23,513-15-7	－	－	－	－	－	－
Alisol B,23-acetate	19,865-76-0	＋	－	－	－	－	－
Pachymic acid	29,070-92-6	－	－	－	－	－	－

### 1.4 Clinical Application and Practice of QFPDD

QFPDD is taken as water decoction, once a day, administrated in the morning and at night separately, 40 min after meals and total of three doses as a course of treatment (Jiang and Chen, 2020). If possible, half a bowl of rice water can be taken after taking the decoction every time, and those suffering from body fluid deficiency can take one bowl of rice water (Tian et al., 2020). Diagnosis and Treatment Program of COVID-19 (Seventh edition) issued by National Health Commission of the PCR has clearly stated that TCM treatment requires syndrome differentiation and treatment based on the local climate characteristics and different physical constitution. QFPDD, as a general prescription, could not take into account individual differences and may bring some related adverse reactions. Common adverse reactions of QFPDD include nausea and vomiting, dizziness, dermatitis, etc. Wang et al. (2020b) collected information about the entire diagnosis and treatment of 98 confirmed cases of COVID-9 treated with QFPDD in Sichuan province, and found that during the course of QFPDD treatment, four patients had nausea and vomiting, two patients had dizziness, one patient had a rash, and the incidence of adverse reactions was 7.14% (Wang et al., 2020b). In addition, Hu et al*.* revealed the observation on clinical effect of Qingfei Paidu granules in the treatment of 76 confirmed cases of COVID-9 in Hubei province, and found that during the course of Qingfei Paidu granules treatment, two patients had mild diarrhea, one patient had nausea and vomiting, one patient suffered from pruritus, and the incidence of adverse reactions was 5.26%, but above adverse reaction symptoms were mild and disappeared without special treatment (Hu et al., 2020). As shown in Table 6, some clinical observation of Qingfei Paidu prescription with different dosage forms in the treatment of COVID-19 indicated that QFPDD could effectively improve the symptoms and the effective rate is above 80%. The specific clinical indicators of TCM syndromes and main laboratory indices and safety observation which reflect the efficacy of QFPDD are shown in Table 7 and Table 8, respectively. For each course of treatment, clinicians should objectively evaluate the efficacy and actual adverse reactions of QFPDD to adjust the prescription appropriately.

**TABLE 6:** Observation on clinical effect of Qingfei Paidu prescription with different dosage forms in the treatment of COVID-19.

No	The number of cases	Pharmaceutical dosage form	Course of treatment	Cure rate (%)	Total effective rate (%)	Province	References
**1**	76 cases	Granules	5 days as a course of treatment, three courses of treatment	65.79%	88.16%	Hubei province	Hu et al. (2020)
**2**	98 cases	Decoction	3 days as a course of treatment, three courses of treatment	41.13%	92.09%	Sichuan province	Wang et al., 2020b
**3**	30 cases	Decoction	3 days as a course of treatment, three courses of treatment	NA	83.335	Hubei province	Li et al., 2020b
**4**	151 cases	Mixture	3 days as a course of treatment, three courses of treatment	43.70%	90.07%	Sichuan province	Lai et al. (2020)
**5**	108 cases	Decoction	3 days as a course of treatment, three courses of treatment	NA	91.67%	Hubei province	Meng et al. (2020)
**6**	214 cases	Decoction	3 days as a course of treatment, three courses of treatment	NA	90%	Shanxi, Hebei, Shaanxi, Heilongjiang province	General Office of National Health Commission, State Administration of Traditional Chinese Medicine (2020)

**TABLE 7:** Clinical symptom rating scale of TCM syndromes of COVID-19.

Primary symptoms	Normal (0 point)	Slight (2 points)	Medium (4 points)	Severe (6 points)
Fever	≤37.2°C	37.2°C–38.2°C	38.3°C–39.0°C	>39.0°C
Cough	None	Occasionally, with a single cough	Often, but does not affect work and rest	Cough frequently with more than one cough, cause vomiting, affects work and rest
Asthma	The respiration is stable and the frequency is within the normal range of the corresponding age	Exceeding the upper limit of the normal value of the corresponding age (≤10 times/min), there is no flaring of nares and three concave sign	Exceeding the upper limit of the normal value of the corresponding age (11–20times/min), and/or intermittent wheezing, flapping of nasal wings, three concave sign	Exceeding the upper limit of the normal value of the corresponding age (≥21times/min), and/or continuous wheezing, flaring of nares, three concave sign
Expectoration	None	There is an occasional sound of phlegm in the throat and a small amount of sputum	The phlegm sound in the throat is hissing and the phlegm is yellow	There is a roar of phlegm sound in the throat and a large amount of yellow-phlegm
Nasal obstruction	None	Occasionally. It doesn’t affect breathing through the nose	Patients often have the nasal obstruction during the day	Obvious nasal obstruction patients have to breathe through the mouths
Nasal discharge	None	Occasionally	Patients have runny nose in the morning and at night	Continuously
Dry mouth	None	Occasionally	Sometimes	Continuously
Pharyngalgia	None	Slightly	Dry pain, pain when swallowing	Burning pain, sharp pain when swallowing
Hypodynamia	Normal	Slightly	Obvious	General weakness
Anorexia	Normal	Poor appetite	Loss of appetite	The appetite is extremely poor, or the patients refuse to eat
Diarrhea	None	Less than 3 times a day Loose stool	Three to six times a day Loose stool	More than 7 times a day The stool is watery
Secondary symptoms	**Normal (0 point)**	**Slight (1 point)**	**Moderate(2 points)**	**Severe (4 points)**
Complexion	Normal	Flushing of face and lusterless complexion	Flushing of face and dim complexion	Pallor and dim complexion
Palpitation	None	Mildly	Sometimes	Continuously
Abdominal distension	None	Occasional abdominal distension or postprandial abdominal distension	Abdominal distension is severe, up to 6 hours a day	Abdominal distension all day long
Aversion to cold	None	Slightly	Moderately	Shivering
Cyanosis	None	Slight cyanosis [*P*(O_2_)50 mmHg–80 mmHg,SaO_2_ 80%–90%]	Moderate cyanosis [*P*(O_2_)30 mmHg–50 mmHg,SaO_2_ 60%–80%]	Severe cyanosis [*P*(O_2_)<30 mmHg,SaO_2_ <60%]
Hyperhidrosis	None	Usually the skin is slightly moist or occasionally hot and sweating	Usually the skin is moist, sweating if you move a little; hectic fever on the chest and back, sweating repeatedly	Sweat usually and sweat like washing with moving
Short breath	None	Slightly	Shortness of breath increases after exercise	Obviously affecting work and daily life
Insomnia	Normal	Difficulty falling asleep	Difficulty falling asleep, sleep lightly	Hard to sleep
Urination	Normal	Slightly yellow	Dark yellow	Dark urine
Tongue manifestation	**Normal (0 point)**		**Abnormal (2 points)**	
Tongue property	Light red tongue		Red or dark-red tongue, or with ecchymosis, or prickly tongue	
Coated tongue	The tongue coating is thin and white		Tongue coating is yellow, thick, greasy, etc.	
Pulse	**Normal (0 point)**		**Abnormal (2 points)**	
Pulse	Normal pulse		Irregular-rapid pulse, irregularly intermittent pulse, regularly intermittent pulse, etc.	

**TABLE 8:** The main laboratory indices and safety observation in the treatment of COVID-19.

Detection of laboratory indices	References	Detection of laboratory indices	References	Safety observation	References
White blood cell count (WBC)	Hu et al. (2020), Wang et al., 2020b, Meng et al. (2020)	Lactate dehydrogenase (LDH)	Wang et al., 2020b	Throat swab nuclei cacid detection	Hu et al. (2020), Wang et al., 2020b
Lymphocyte percentage (LYMPH%)	Hu et al. (2020), Wang et al., (2020b), Meng et al. (2020)	Creatine kinase isozyme (CK-MB)	Wang et al., 2020b	Chest computed tomography	Hu et al. (2020), Wang et al., 2020b
Neutrophil percentage (NEUT%)	Hu et al. (2020), Wang et al., (2020b), Meng et al. (2020)	Creatine kinase (CK)	Wang et al., 2020b	Blood biochemistry	Hu et al. (2020), Wang et al., 2020b
Aspartate aminotransferase (AST)	Wang et al., 2020b, Meng et al. (2020)	C-reactive protein (CRP)	Hu et al. (2020), Wang et al., 2020b, Meng et al. (2020)	Electrocardiogram	Hu et al. (2020), Wang et al., 2020b
Erythrocyte sedimentation rate (ESR)	Hu et al. (2020), Wang et al., 2020b	Procalcitonin (PCT)	Hu et al. (2020), Wang et al., 2020b	Observation of adverse reactions	Hu et al. (2020), Wang et al., 2020b
Albumin (ALB)	Hu et al. (2020), Meng et al. (2020)	D-dimer (D-dimer)	Hu et al. (2020), Wang et al., 2020b		
Urea (UREA)	Hu et al. (2020), Wang et al., 2020b, Meng et al. (2020)	Alanine aminotransferase (ALT)	Hu et al. (2020), Wang et al., 2020b, Meng et al. (2020)		
Creatinine (CREA)	Hu et al. (2020), Wang et al., 2020b, Meng et al. (2020)				

If the patient does not have a fever, the dosage of raw gypsum should be reduced, otherwise, the dosage of raw gypsum should be increased. If the symptoms are improved but not cured, the second course should be added. If the patient has other basic diseases, the second course of the prescription shall be modified according to the actual situation. (You et al., 2020). If the symptoms disappear, the patients can stop taking the medicine in the second course of treatment. For patients with obvious deficiency of spleen *Yang*, 15 g of raw gypsum can be used in the prescription; for patients with the deficiency of stomach *Yin*, the method of nourishing *Yin* and eliminating dampness can be followed for empirical treatment and for those with excessive sweating, high blood pressure, palpitation, and insomnia, the dosage of the prescription can be appropriately reduced, or the dosage of yam can be increased. In the case of hepatic insufficiency, clinicians should analyze the causes of hepatic insufficiency, stop taking drugs if necessary, or add liver protection therapy (Dong et al., 2020; Lai et al., 2020). As for the dosage of *Herba Asari*, QFPDD is used up to 6 g, although it does not follow “the dosage of *Herba Asari* is not more than 5 g”, it is still in the range of commonly used clinical dosage and its fluctuation, which is more suitable for the patients with cold-dampness-yang injury and severe deficient cold. For those with severe heat and dampness, the dosage of *Herba Asari* should be reduced as appropriate (Liu et al., 2020a). Some scholars also have recommended the modified QFPDD combined with western medicine such as alpha-interferon, oseltamivir, chloroquine phosphate, arbidol, ribavirin in the treatment of COVID-19, and found that it was more effective than the treatment of western medicine alone, which could significantly shorten the patient’s hospitalization time, the time of clinical symptom improvement and the time of lung CT improvement (Fang et al., 2020; Li et al., 2020b; Yang et al., 2020b).

## 2 Conclusion and Perspectives

COVID-19 is a new type of infectious disease. Western medicine mainly focuses on symptomatic relief. TCM has been applied for treating epidemics for thousands of years, and many clinicians have conducted in-depth research on COVID-19 etiology, pathogenesis, and syndrome differentiation. Since TCM played a huge role in the treatment of SARS in China in 2003, the National Health Commission and the National Administration of TCM jointly issued the “New Coronavirus Infection Pneumonia Diagnosis and Treatment Program (Fourth, Fifth, Sixth, Seventh and Trial Eighth edition)”, which advocated the integration of Chinese and Western medicine, strived to shorten the course of the disease, improve clinical efficacy and reduce the incidence and mortality of critically ill patients (Lu and Lu, 2020; Xie, 2020).

In the process of the treatment of COVID-19, under the guidance of TCM theory, based on clinical practice and patient-oriented principle combined with data mining and basic research of modern biology and pharmacology, China established treatment methods for different stages and syndromes in different regions by systematically sorting out several classic and effective prescriptions and quickly put them into the clinical application (Zhang et al., 2020). Given the current epidemic situation of COVID-19, early intervention of TCM has played an important role in this epidemic control. Chinese and western advantages complement each other, which has a definite curative effect in reducing fever and other symptoms, controlling disease progression and reducing complications. QFPDD was selected and recommended by the National Administration of TCM as a general prescription for treating different stages of COVID-19. QFPDD is combined with multiple prescriptions and has the properties and flavors of pungent-warm and pungent-cool, aiming at the pathogenesis of COVID-19, including cold, dampness, heat, toxin, and deficiency (Chen et al., 2020b). QFPDD has the functions of dispelling cold and dampness, eliminating heat and turbidity, promoting and nourishing lung and spleen, detoxifying and removing pathogenic factors, etc. Modern pharmacologic studies have also confirmed the anti-inflammatory, antiviral and immunological functions of QFPDD which is attributed to the multi-component, multi-target, and multi-pathway characteristics of TCM. QFPDD is also a widely accepted prescription for treating COVID-19 based on its successful and effective clinical observations. The successful use of QFPDD in this novel viral pneumonia epidemic has confirmed the advantages of TCM in treating emergencies. However, at present, the mechanism of QFPDD is still unclear. It is necessary to further comprehensively evaluate the efficacy and safety of QFPDD and clearly explain the complex mechanisms of QFPDD in the treatment of COVID-19 through systematic reviews and meta-analysis (Gao et al., 2020). Currently, there is a lack of extended research with sufficient breadth and depth and the current research has just focused on QFPDD TCM theory, clinical experience, network pharmacology, etc., with only a small number of clinical research samples. In the follow-up research, it is not only essential to carry out more comprehensive chemical composition characterization, pharmacokinetic and pharmacodynamic studies *in vitro* and *in vivo*, but also extended clinical data should be evaluated to elucidate the material basis and systematically explain the effectiveness of QFPDD against COVID-19, and further provide a theoretical basis for the clinical scientific and rational application of QFPDD in the prevention and clinical treatment of COVID-19.

## Author Contributions

RW, YM, RQW, LP, GL, and SJY conceived and designed the review; QS, QGP, LD and MM reviewed the literature; RW and YM wrote the manuscript.

## Funding

This work was supported by National Administration of Traditional Chinese Medicine (Grant NO. 2020ZYLCYJ02?3); Sichuan Administration of traditional Chinese Medicine [(Grant NO. 2020yj018) and (Grant NO. 2020yj024)]; the fellowship of China Postdoctoral Science Foundation (Grant NO. 2020M683365); the National Traditional Chinese Medicine Clinical Research Base (Grant NO. (2020)33). The authors are sincerely thankful to all individuals who were involved in this study.

## Conflict of Interest

The authors declare that the research was conducted in the absence of any commercial or financial relationships that could be construed as a potential conflict of interest.
