# Supplementary figures and images for: Research Advance on Qingfei Paidu Decoction in Prescription Principle, Mechanism Analysis and Clinical Application
Source: Front Pharmacol. 2021 Jan 27;11:589714. doi: 10.3389/fphar.2020.589714 (PMC7873690; doi:10.3389/fphar.2020.589714)

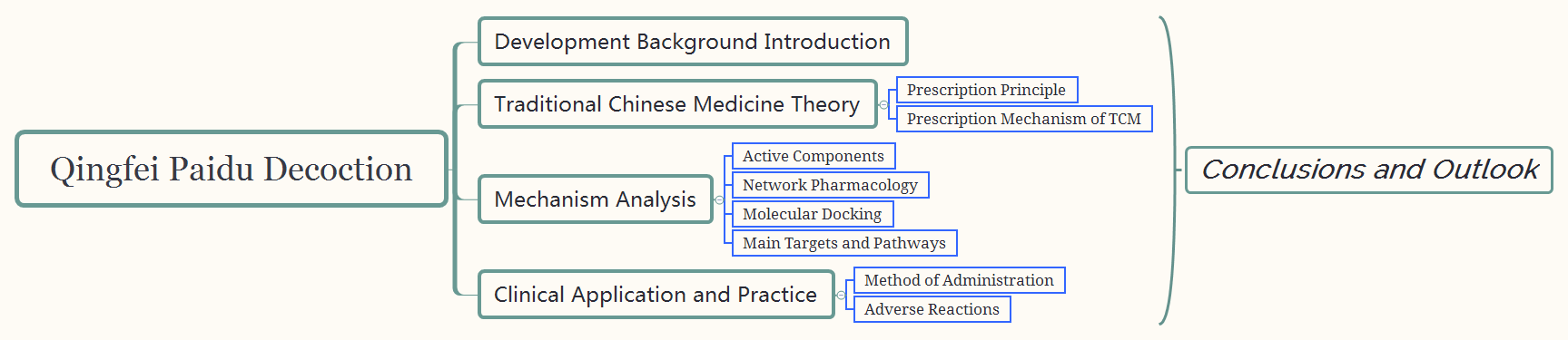

Supplement: Supplementary file 1 [file image1.png]
